# Migrasome: a new functional extracellular vesicle

**DOI:** 10.1038/s41420-023-01673-x

**Published:** 2023-10-18

**Authors:** Xide Zhang, Liuhuan Yao, Yuanyuan Meng, Bailong Li, Yanyong Yang, Fu Gao

**Affiliations:** 1 Department of Radiation Medicine, Faculty of Naval Medicine, Naval Medical University, 200433 Shanghai, P. R. China; 2 Naval Medical University, Department of Traditional Chinese Medicine, Affiliated Hospital 1, 200433 Shanghai, P. R. China

**Keywords:** Cell migration, Cancer therapy

## Abstract

Migrasome is a novel cellular organelle produced during cell migration, and its biogenesis depends on the migration process. It is generated in a variety of cells such as immune cells, metastatic tumor cells, other special functional cells like podocytes and cells in developing organisms. It plays important roles in various fields especially in the information exchange between cells. The discovery of migrasome, as an important supplement to the extracellular vesicle system, provides new mechanisms and targets for comprehending various biological or pathological processes. In this article, we will review the discovery, structure, distribution, detection, biogenesis, and removal of migrasomes and mainly focus on summarizing its biological functions in cell-to-cell communication, homeostatic maintenance, embryonic development and multiple diseases. This review also creates prospects for the possible research directions and clinical applications of migrasomes in the future.

## Facts

1. Migrasome is a newly discovered extracellular vesicle, whose production depends on cell migration.
2. The formation of migrasomes is closely associated with TSPANs and integrins, and is regulated by various factors.
3. Migrasome plays an important role in various fields including cell-to-cell communication, homeostasis maintenance, embryonic development, various diseases occurrence, progress and diagnosis, etc.
4. Tumor cells may be the main field of future migrasome research due to their strong invasiveness and metastasis.

## Questions

1. Who will mediate the recognition and uptake of migrasomes by later cells?
2. Can migrasome also mediate the quality control of other organelles to alleviate cell stress?
3. Does the decrease in tumor cell adhesion have an impact on migration generation?
4. Is migrasome a more economical option for cells compared to other extracellular vesicles?

## Discovery and structure of migrasome

In 1963, Taylor and Robbins [1] recorded the retraction fibers in the rear of migrating cells through optical microscope and transmission electron microscope. For a long time, researchers have focused on the junction of retraction fibers and cell membrane. The characteristics of retraction fibers have been neglected. Until 2015, Ma et al. [2] found migrasomes at the terminal or cross connection of retraction fibers. Migrasome is a novel class of extracellular vesicles (EVs) dependent on cell migration. The characteristics of migrasomes are different from those of previously discovered Evs [3–7], which seems to indicate their unique functions (Table 1).

**Table 1:** Characteristics of migrasome.

Items	Characteristics	References
Size	500 nm to 3000 nm	[2, 12, 54, 74]
Shape	Round or oval	
Origin	Migratory cells	
Distribution	The rear of cells	
Location	The terminal or cross connection of retraction fibers	
Content	Proteins, RNAs, organelles, and numerous smaller vesicles	
Stability	Persisting after disappearance of the retraction fibers	
Move	Almost not	

## Distribution and detection of migrasome

### Distribution of migrasome

Migrasome is widely distributed [2, 8–17]. So far, traces of migrasomes have been found in human, mouse, rat, and zebrafish [18]. It is common in cells with strong migration ability, such as immune cells and metastatic tumor cells, etc. For example, receptor activator of nuclear factor κ-B ligand (RANKL) can stimulate murine monocyte-macrophage cell line (RAW 264.7 cells) to differentiate into osteoclast, and induce the formation of migrasomes during differentiation [15]. In addition, migrasomes are usually present in cavities such as alveoli or blood vessels. For instance, within intestine, migrasomes are present in capillaries or lymph capillaries, ileal crypt lamina propria or in connective tissues [16].

### Detection of migrasome

Migrasomes have many characteristic markers [8, 19] (Table 2). Labeled migrasomes can be observed by total internal reflection fluorescence spectroscopy (TIRF) or live cell imaging and so on [19, 20]. Moreover, Jing et al. [21] used single particle tracking of fluorescent artificial antigen to superresolve the membrane fiber network and migrasomes. At present, there are still many limitations in using fluorescent labeled protein to detect organelles, such as long experiment period, poor universality and potential influence of marker protein itself, etc. It is still necessary to find a rapid, easy, safe and non-interfering method for detection of migrasomes. To achieve this goal, it is imperative to separate and purify the migrasome. Initially, researchers isolated migrasomes by density gradient centrifugation [2, 10]. First, removing cell bodies and large cell debris by centrifugation at 1000–4000 × *g*. After this, the supernatant is collected and centrifuged at high speed (20,000 × *g*) to pellet the migrasomes. Subsequently, the migrasome-containing pellet is resuspended and subjected to density-gradient centrifugation at 150,000 × *g*. The final ultracentrifuged product is divided into several fractions, and the migrasome fraction is washed in an equivalent volume of PBS and spun down at 20,000 × *g* to obtain the migrasome pellet [22]. So far, it is still the main method for the purification of migrasomes [22, 23], similar to EVs. The difference lies in: (1) because of the migration dependence of migrasome, cells need to be cultured in dishes coated with special coatings; (2) a higher centrifugal speed is required [23–25]. At present, there is no specific separation method for migrasomes, which still needs to be explored.

**Table 2:** Markers of migrasome.

Markers	Detection methods	References
Integrins	Mass spectrometry analysis	[19]
TSPAN4	GFP labeling and confocal microscope	[2]
NDST1, PIGK, CPQ, EOGT	Live cell imaging	[8]
WGA	Fluorescently tagged WGA probe	[75]

## Biogenesis and removal of migrasome

### Biogenesis of migrasome

#### Formation of migrasome

Previous studies have shown that the formation of migrasomes is closely associated with various molecules, such as integrin, tetraspanins (TSPAN) and cholesterol [19, 26]. It is believed that integrins may play a dual role in migration: (1) making cells migrate; (2) promoting migrasome formation. The aggregation of integrins at the bottom of the migrasome formation site and adhesion to the extracellular matrix may be the initiation of migrasome [19].

TSPAN is another the most powerful promoter of migrasome formation, enriched at the top of migrasomes and retraction fibers [27]. The concentration of TSPANs in migrasome membrane increases with time [28]. On the retraction fiber membrane, TSPAN forms tetraspanin-enriched microdomains (TEMs) with cholesterol and other molecules, which subsequently compose tetraspanin-enriched macrodomain (TEMA) [26]. TEMA in turn changes its shape and becomes a migrasome [26]. The entire process is divided into two sequential steps: formation of local swellings on the tubular retraction fibers, and stabilization of these swellings through TEMAs [29]. The above assumption raises another question: who initiated the local expansion of retraction fibers? Latest research has found that sphingomyelin synthase 2(SMS2) assembles into immobile foci, which enters retraction fbres, where they become migrasome formation sites [30]. Ceramide is converted to sphingomyelin(SM) on the SMS2 foci, which may trigger the growth phase of migrasome formation by promoting the assembly of TEMs through its interaction with cholesterol [30]. The mechanism by which SMS2 foci determine the site of migrasome formation has not been clarified.

It is worth noting that the migrasome can still exist stably after breaking away from the retraction fibers. Why does the detached migrasome hardly move? What is the role of integrins and ECM proteins in the stable state of migrasomes? Which molecules mediate the enrichment of integrins or TSPAN? The mechanism of migrasome formation still needs further exploration.

#### Regulation of migrasome formation

The regulation of migrasome formation is also an important component. Fan and colleagues reported that migration persistence and speed are critical cell migration parameters for migrasome formation [31]. In addition to kinetic parameters, other physical factors such as temperature and pH may also affect the formation of migrasome.

The molecular regulation of migrasome formation is not well understood. Studies have shown that some viruses [32], drugs [33], cytokines [34], peptides [35] or genes [36] can regulate the formation of migrasomes. In addition, the photosphatedylinositol (4,5)-bisphotosphate-Rab35 axis is also one of the important pathways [37, 38]. However, the recruitment principles of relevant factors and the universality of the mechanisms are still unknown. There are still many unsolved mysteries about the formation and regulation of migrasomes. More mechanisms require further investigation.

### Removal of migrasome

**Fig. 1:**
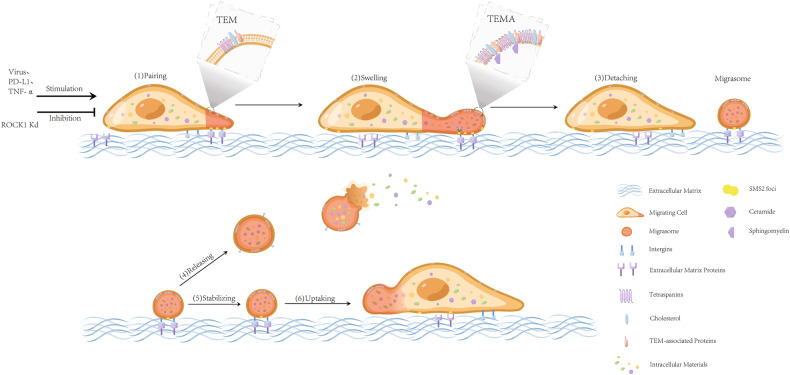
Biogenesis and removal of migrasome. The integrins of migrating cells bind specifically to extracellular matrix proteins. The intracellular substances are transported to the migrasome site along the retraction fibers, and migrasome gradually expands. TEMs form TEMAs, altering the physical properties of the membrane and maintaining morphology. The disconnected migrasome is eventually ingested by other cells or ruptured.

The average presence length of the migrasomes is approximately 400 minutes [2]. They could be divided into two outcomes: some will rupture and release contents called “migracytosis”; others can be absorbed by other cells [2]. (Fig. 1).

There is no study demonstrating the existence of a special migrasome removal process. However, as a novel cellular organelle, migrasome contains a lot of intracellular substances. The release of them via migracytosis may affect homeostasis. In order to maintain the balance, we speculate that specific migrasome clearance pathways should exist. Being absorbed by other cells may be one of the clearance routes. But whereabouts of migrasomes not ingested by other cells as well as their content are unclear. Whether will they enter the circulation system? Are there specific cells responsible for “processing”? All of these will require further investigation.

From formation to clearance, the “lifetime” of the migrasome is complicated. The mechanism has not been fully defined. And the formation mechanism of vesicle structure within the migrasomes is either unclear.

## Function of migrasome

**Fig. 2:**
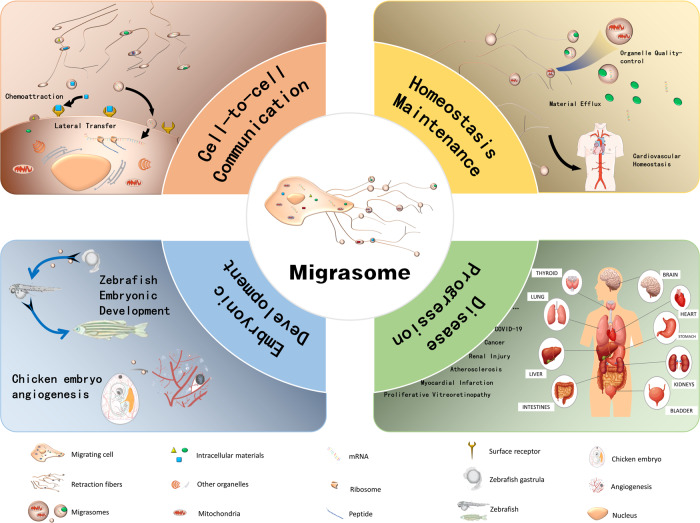
Functions of migrasome. Migrasome plays an important role in various fields including cell-to-cell communication, homeostasis maintenance, embryonic development, various diseases occurrence, progress and diagnosis, etc.

Recent researches showed that the migrasome plays an important role in various fields including cell-to-cell communication, homeostasis maintenance, embryonic development, various diseases occurrence, progress and diagnosis, etc. (Fig. 2). At present, the known functions of migrasomes are various, and we summarize them as follows.

### Migrasomes and cell-to-cell communication

Mediation of material exchange and information transfer among cells may be the primary function of migrasomes. Zhao et al. [39] used two-photon synthetic aperture microscope to directly observe the intercellular communication through migrasomes. Different from thin cellular bridges [40, 41] and gap junctions [42, 43], migrasomes present a novel mean for cell–cell communication [44, 45] and lateral transfer of cellular contents [46]. For example, DCs can utilize migrasomes to transfer cytosolic components (such as antigens and chemokines) to other DCs [21]. Following traumatic brain injury (TBI), neutrophils in the peripheral immune system can deliver signals directly to microglia through migrasomes [39]. Zhu et al. [46] revealed that migrasomes contain mRNAs and proteins, which can be laterally transferred into recipient cells. The mRNAs are then translated into proteins which can functionally modify the recipient cell [46]. These mRNAs and proteins may be the executors of the migrasome function, but the mechanism of how cells transport these specific molecules into migrasomes remains to be determined. Migrasome-mediated intercellular information transmission is extremely special because it only exists in migrating cells. The information related to cell migration may be the main content of migrasomes, such as factors of proper polarization and migration direction. It is noteworthy what role retraction fibers plays in this process. Do the retraction fibers play the role of regulating the action route as the track? Relevant mechanisms need to be explored.

### Migrasomes and homeostatic maintenance

The migrasome plays important roles in homeostatic maintenance. They participate in the balance of intracellular substances, the treatment of damaged organelles, and the homeostasis of multiple systems. For example, migrasome is pivotal in endovascular homeostasis [16]. TSPANs, the important structural molecule of the migrasome, are widely expressed in the cardiovascular system, and involved in the physiological and pathological processes associated with thrombosis, hemostasis, angiogenesis, vascular injury (including vascular cell migration), and even heart development [16]. In addition, Schmidt-Pogoda et al. had identified DNA-interacting proteins in migrasomes from the infarcted brain parenchyma of mice [17], suggesting that migrasomes may affect the homeostasis of the central nervous system, but further research is lacking. Gustafson et al. [44] found that NCCs (neural crest cells) release exosomes and deposit migrasomes, which is necessary to maintain directional movement of polarized cranial migratory NCCs. Once again, it confirms the close correlation between migrasomes and the central nervous system.

Apart from macroeconomic regulation, migrasomes are also crucial for cell homeostasis. As a physiological process mediated by migrasome, mitocytosis regulates the transfer of intracellular substances to extracellular, which is of great significance for the maintenance of cell homeostasis, the communication between cells and the exchange of substances between cells and extracellular matrix [9]. For instance, migrasome can mediate cell mitochondrial quality control [9, 47] and take part in cholesterol metabolism [48, 49] through mitocytosis. There are still many issues regarding mitocytosis. Do other organelles have similar quality control mechanisms? Can normal organelles be transferred between cells through mitocytosis? Is dealing with dysfunctional organelles the common mechanism for maintaining cell survival? If mitocytosis is part of a broader cellular process that protects the integrity of organelles, are there regulatory mechanisms? Are migrasomes involved in the metabolism and homeostasis of other substances? If cells can use migrasomes to shed unwanted substances, does it also mean that they can bring essential materials? Are migrasomes “supply stations” or “garbage can” on the “track” of cell migration? These all require further research.

### Migrasomes and embryonic development

Migrasomes often contain a variety of inducible factors [50], which mediate the biogenesis of organs. In 2019, Jiang et al. [10] found that the migrasomes rich in chemokine Cxcl12a were enriched in the cavity below the embryonic shield of the zebrafish gastrula and affected the establishment of the body axis and organ morphogenesis of zebrafish by regulating Cxcl12a signaling pathway [10]. Similarly, Zhang and workmates [51] revealed that monocyte migrasomes rich in pro-angiogenic factors, such as VEGFA and CXCL12, promote capillary formation and monocyte recruitment in the chorioallantoic membrane of chick embryos [51]. Furthermore, migrasomes were shown to act as a chemotactic cue for monocytes, and more monocytes are recruited through the feedforward loop to promote angiogenesis [52].

In addition to zebrafish and chicken embryo, there are also studies about the effects of migrasomes on human organogenesis. Deniz and coworkers [53] have detected stromal cell-derived factor1 in the migrasome of plastic-adherent mesenchymal stromal cell (MSCs) isolated from human bone marrow, which can attract KG-1a leukemic cells and primary CD34+ hematopoietic progenitors. Migrasomes support a new mechanism for communication between MSCs and hematopoietic derived cells, suggesting a possible connection with bone marrow biogenesis. Are there similar migrasome-dependent phenomena during embryonic or organ development in other animals? This is an important direction for the research of migrasome function. Further studies are needed to decipher all biological aspects of migrasomes in organogenesis.

### Migrasomes and diseases

Compared to the aforementioned physiological implications of migrasomes, the clinical application of migrasome modulation represents a path not well explored so far [54], though studies on the relationship between migrasomes and diseases have never stopped. Up to now, multiple studies have pointed out connections between migrasomes and various diseases.

#### Migrasomes and cardiovascular disease

As mentioned earlier, migrasome and TSPAN are closely related to cardiovascular homeostasis [9]. Homeostasis and disease are like two sides of yin and yang. This undoubtedly implies a myriad of connections between migrasomes and cardiovascular diseases. The recent study [55] has shown that TSPAN4 expression is highly associated with atherosclerosis regression-related macrophages, intraplaque hemorrhage as well as ruptured plaques, and is upregulated in spontaneous myocardial infarction (MI) and inducible MI mice model. So, migrasome may be a novel potential target of the regression macrophages response to atherosclerosis progression [56].

#### Migrasomes and urinary diseases

Migrasomes occupy a central position in the development of some diseases [57]. One example is that migrasome is a potential biomarker for diagnosis of podocyte stress nephropathy induced by lipopolysaccharide (LPS), etc. [12], which seems to be more sensitive and reliable indicators than proteinuria [13]. However, there is still a distance from clinical application, and many problems still need to be solved. Can the urinary migrasome serve as a marker of renal function? The number of migrasomes in blood is larger than urine and they act as an important part to vascular regulation. Further researches are needed to investigate whether blood migrasomes can be used as a marker of diagnosis in the future.

#### Migrasomes and neurological diseases

Antje et al. [17] found migrasomes rich in neuronal fragments near atrophic neurons, suggesting that they may mediate the clearance of damaged neurons or exacerbate neuronal atrophy. In vitro, sodium chloride induction experiments have also demonstrated that high salt environment can increase the formation of migrasomes by microglia and promote the pro-inflammatory polarization of microglia [17]. Although the mechanism is unclear, a migrasome mediated mode of damaged cell clearance, which does not rely on apoptosis, seems to be faintly visible. Whether this pathway involves in the progression of nervous system diseases remains to be explored. The latest research has found that amyloid beta protein 40 (Aβ40) stimulates macrophage lineage cells to overproduce the migrasome containing complement activation-related molecule CD5L, thus further promoting complement-dependent blood–brain barrier damage [25]. The complement-related processes mediated by migrasomes suggest their multifaceted involvement in the body’s immune system. At the same time, it also provides new ideas and directions for the study of migrasome functions.

#### Migrasomes and infection

Regulating immune response by transmitting pathogens is one of the important functions of classical Evs [58]. So, what about migrasomes? Does migrasome-mediated lateral transfer promote virus invasion or immune activation? Koupenova et al. [11] revealed that platelets internalize SARS-CoV-2, and then viral internalization leads to rapid digestion, programmed cell death and release of migrasomes. The contents of these migrasomes can be highly thrombogenic or pro-inflammatory, and may lead to immune activation disorders [11]. These indicated that migrasomes might contribute to thromboinflammation in COVID-19 [18]. In addition, the latest researches suggest that both herpes simplex virus type 2 (HSV-2) and vaccinia virus (VACV) can be transmitted through migrasome-pathway [23, 59]. Virus infection itself is also a trigger for the generation of migrasomes, and the relationship between them still needs further research.

#### Migrasomes and tumor

Tumor cells have high invasiveness, metastasis and strong migration ability, which are potential sources of migrasomes. Besides, TSPAN4 was highly correlated with tumor-associated macrophages [55]. Previous studies have suggested that migrasomes may have an important effect on the occurrence and development of cancer and might be new targets for cancer treatment. For example, Qin et al. [60] carried out pan-cancer analysis through a bulk omics research and single cell sequencing validation, and then identified migrasome-related genes as a potential immunotherapeutic target and estimated high expression of migrasome contributed to poor prognosis. Zheng and colleagues [61] further found that TSPAN4 expression is associated with pan-cancer, especially in Glioblastoma multiforme and Brain Lower Grade Glioma. Analyzing the immune subtypes of samples from different tumor types revealed differences in the expression of TSPAN4, and TSPAN4 gene may participate in tumor progression through different mechanisms [62].

As a new target for disease treatment, the intervention of migrasomes may save dying patients’ lives and improve their prognosis. In 2022, Cheng et al. [63] selected several anti-migration nanoparticles, and discovered that they closely bound to retraction fibers (RFs) and migrasomes through intensive interactions with lipid rafts/caveolae substructures. Considering the widely established correlations between multiple nanoparticles and lipid rafts/caveolae [64–66], this may be a general mechanism. Blocking the intrinsic migration promoting effect of migrasomes through nanoparticles may be a new strategy for anti-metastasis nanotherapy. Their study provides new insights into the role of migrasomes in tumor prognosis and immunotherapy [60].

#### Migrasomes and other diseases

In addition to the aforementioned, diseases in other fields also have the presence of migrasomes. Wu et al. [67] found that TSPAN4-positive migrasomes play a pivotal role in retinal pigmented epithelium cells (RPE) activation and proliferative vitreoretinopathy (PVR) progression. Migrasomes derived from RPEs stimulated by PVR microenvironment can be internalized by other RPEs, which leads to the improvement of migration and proliferation ability, forming positive feedback [67]. Targeting TSPAN4 or blocking migrasome formation might be a new therapeutic method against PVR [67]. Cell migration exists in every corner of the human body, which undoubtedly provides a broad space for disease-related migration research.

## Future perspectives of migrasome

Migrasomes can import cellular material through new avenues, providing new protein targets, insights, and avenues for researchers to utilize and ultimately advance EV-based research [50]. Although researches on functions of migrasome is in full swing, one of the most fundamental problems about migrasome has not been solved. Who are the “ancestors” of migrasomes? Is there a similar process in unicellular organism or others? Compared to “boisterous” EVs such as exosomes, are relatively static migrasomes more directional? Conducting extensive and profound research on diverse life forms can help answer this interesting question. While the mechanisms of migrasome formation are still being elucidated, they have been shown to be enriched for tetraspanins and cholesterol [68]. At present, the control mechanisms and regulatory pathways related to migrasomes are still vague. What does the position of migrasomes along retraction fibers depend on? Is there a planning or periodicity in the formation of migrasomes? Is there a difference in the ability of divers migrating cells to produce migrasomes? What is the distinctness in migrasomes generated by the same cell at different stages? It should be also considered that the exchange of information between migrasomes and other membranous organelles, such as exosomes, might also influence the functions of migrasomes [69]. In view of the special formation pattern of migrasome, the role of cytoskeleton in it cannot be ignored. Understanding the mechanism underlying migrasome biogenesis should be the priority, otherwise it is going to be very difficult to build a convincing case for functional study [70].

Besides the functions mentioned above, what other biological or pathological processes are migrasomes involved in? Many immune cells migrate during their development and in immune responses and they secrete a large array of signaling molecules [71]. This seems to be related to migrasomes. Similarly, many other processes, including tumor metastasis, angiogenesis, wound healing and tissue regeneration, require cell migration and secretion [71]. What kinds of roles migrasomes play among them deserves further exploration.

Migrasomes often contain different numbers of smaller vesicles [72]. However, few studies about the internal structure of the migrasome have been reported. Ma et al. [73] reported the discovery of EV-like migrasome-derived nanoparticles (MDNPs) that are produced by migrasomes via self-rupture or through a process similar to cell plasma membrane budding, which are loaded with a large number of miRNAs different to those found in migrasomes and EVs. What are the vesicles inside migrasomes? Are these small vesicles transferred from cytoplasm to migrasome? Are they produced during the migrasome formation? Are they also a novel type of extracellular vesicle? Is the material contained inside them the specific molecules that really works? Is there still a secondary process of internal vesicle formation or clearance? Is the migrasome the ultimate functional executor or the “transfer station” for the internal vesicles? Migrasomes can still exist stably for a period of time after it breaks away from the retraction fibers. What changes have taken place in MDNP during this period? Perhaps in the future, researchers can give answers.

Compared with most normal cells, the migration ability of cancer cells is significantly improved in conjunction with their aggressiveness, and they are more likely to produce migrasomes. However, cancer cells have a reduced adhesion capacity. Does this have an impact on its ability to produce migrasomes? Do cancer cells have a similar quality control process as “mitocytosis”? What roles do migrasomes play in the formation of the tumor microenvironment?

At present, there are still some difficulties in the study of migrasomes. First, there are numerous types of cells capable of producing migrasomes. This provides not only broad prospects for researches, but also brings a lot of problems for researchers. Perhaps researchers can make breakthrough findings by studying migrasomes produced by a kind of cells. But it is a greater challenge to summarize the general law through special examples. Why do cells produce migrasomes? Is it the consideration of saving energy? Is it necessary to realize special functions? Is it the driver to maintain homeostasis? Or is it a pathological state of self-saving or self-destruction? The huge application prospects of migrasomes in organ development, homeostatic maintenance, disease diagnosis and disease treatment, are still worth further exploration. In addition, there are few clinical studies about migrasomes. Recent study has demonstrated that migrasomes from bone marrow mesenchymal stem cells mediates secretion of the antibacterial peptide dermcidin and LC3-associated phagocytosis (LAP) of macrophages [24]. Migrasomes may be a promising therapeutic candidate. However, the cell migration dependence of the migrasome may affect its preparation, thereby increasing the difficulty of clinical transformation. With the development of technology and the extensive promotion of multidisciplinary cooperation, as well as the deepening of the research on the generation, transfer and release mechanism of migrasomes, researchers can combine the results with clinical cases to realize the transformation of the theoretical knowledge of migrasomes into the diagnosis and treatment of diseases.
